# Convergent, discriminant, and known groups validity of the Behavioral Assessment Screening Tool (BAST) in chronic traumatic brain injury

**DOI:** 10.21203/rs.3.rs-3909294/v1

**Published:** 2024-02-15

**Authors:** Shannon B. Juengst, Brittany Wright, Leia Vos, Gabriel Rodriguez, Michael Conley, Lauren Terhorst

**Affiliations:** TIRR Memorial Hermann; UT Southwestern Medical Center; CoreWell Health Medical Group; TIRR Memorial Hermann; UT Southwestern Medical Center; University of Pittsburgh

**Keywords:** traumatic brain injury, psychometrics, measurement, neurobehavioral, validity

## Abstract

The Behavioral Assessment Screening Tool (BAST) measures self-reported neurobehavioral symptoms commonly experienced by adults with traumatic brain injury (TBI). To assess the convergent, discriminant, and known-groups validity of the BAST among community-dwelling adults with chronic traumatic brain injury (TBI), we conducted correlation analyses and tests of group differences with previously validated symptom measures in two samples (n = 111, n = 134). Measures used for comparison were: Patient Health Questionnaire (depression), Generalized Anxiety Disorder-7 (anxiety), Positive and Negative Affect Schedule, Frontal Systems Behavior Scale (Executive Dysfunction, Apathy, Disinhibition), Modified Fatigue Impact Scale, PROMIS Fatigue, Aggression Questionnaire (anger, hostility, physical and verbal aggression), and Alcohol Use Disorders Test (alcohol misuse). BAST subscales had stronger correlations with measures of similar (|r|=.602-.828, p < .001) and related (|r|>.30, p < .001) constructs and weaker correlations (|r|<.300) with measures of dissimilar/unrelated constructs, supporting hypotheses of convergent and discriminant validity, respectively. Statistically significant group differences (p’s < .001) in BAST subscales were found, with large effect sizes (Cohen’s d = 1.2–1.9), for known-groups with moderate-severe depression, moderate-severe anxiety, clinically significant fatigue, problematic disinhibited and frontal-executive behaviors, and alcohol use.

**Conclusions:**

Results support the convergent and discriminant validity of the BAST subscales. The BAST was specifically developed as a self-reported measure for remote symptom reporting, supporting its incorporation into mobile health platforms to improve chronic symptom monitoring in community-dwelling adults with TBI. With further validation research, the BAST could be used for early identification of persons with TBI who could benefit from intervention.

## Introduction

Behavior after traumatic brain injury (TBI) manifests from a combination of premorbid and post-injury factors, including neuropsychological functioning [1, 2], personality traits [3, 4], environmental factors [5, 6], family dynamics [7, 8], and injury characteristics [9], among others [10]. Behavioral problems or symptoms are commonly associated with poor chronic outcomes [11–14]. Managing and reporting of chronic symptoms after TBI requires both awareness and accurate reporting of these symptoms, particularly for neurobehavioral symptoms that fluctuate considerably from day to day [15, 16]. After hospital discharge, patients may have infrequent contact with brain injury specialists, requiring them to recall their symptoms over a long period of time, which presents many challenges. Effective measurement of these symptoms is crucial for the ongoing care of TBI survivors, and technology has helped as a bridge in symptom reporting and tracking [17]. However, before we can effectively leverage technological capabilities, we need symptom measures that are scientifically designed and validated for remote and independent self-reporting after TBI. A well-validated assessment can help clinicians detect symptoms, inform treatment plans, and measure response to intervention.

Based on a conceptual model of behavior after brain injury [10], the Behavioral Assessment Screening Tool (BAST) was developed for this purpose [18, 19], with easy-to-read items measuring frequency of real-world experiences common after TBI. The BAST measures the overlapping influential factors, including emotional state, personal factors, cognitive control, and environmental supports and stressors, that lead to behaviors [10, 20]. The BAST was created based on input from persons with lived experiences and experts in TBI [18] to assess a wide variety of neurobehavioral concerns commonly experienced after brain injury (e.g., aggression, impulsivity/disinhibition, motivational problems, difficulties with planning and problem solving, etc.). The BAST demonstrates strong content validity, as assessed by experts in brain injury rehabilitation and community-dwelling adults with brain injuries and their family members, and through linking the BAST items to the International Classification of Functioning, Disability, and Health [18, 20]. Given the complexities and multiple determinants of behavior, we would expect scales measuring neurobehavioral symptoms, such as the BAST, to be multidimensional. Exploratory Factor Analysis of the BAST resulted in five subscales covering five distinct domains of neurobehavioral function: Negative Affect, Executive Dysfunction, Fatigue, Impulsivity, & Substance Use [19]. The five subscales demonstrate adequate to excellent internal consistency reliabilities, indicating that they each cover a unidimensional construct [19, 21]. Further psychometric validation of the BAST subscales through Rasch Analysis confirmed the unidimensionality of the subscales and indicated excellent item and person separation indices [21].

To further investigate the construct validity of the BAST, our purpose herein was to evaluate the convergent, discriminant, and known-groups validity of the BAST subscales in community-dwelling adults with a history of complicated mild to severe TBI using classical test theory (CTT) methods. Table 1 presents our hypotheses for convergent and discriminant validity evidence for the BAST subscales compared to other measures. We hypothesized stronger correlations between the BAST and similar/related constructs and weaker correlations between the BAST and dissimilar measures. We further hypothesized that the BAST subscale would differentiate between those with probable depression, anxiety, and alcohol misuse and those with clinically significant fatigue, disinhibition, and executive dysfunction.

## Materials and Methods

### Design & Participants

Data from two prospective psychometric studies were combined for these analyses. The first study cohort comprised n=110 community-dwelling adults with a ≥3-month history of complicated mild to severe TBI; the second study cohort comprised n= 135 community-dwelling adults with a ≥3-month history of mild to severe TBI. Detailed methods for the parent studies have been previously published [19,21]. Briefly, participants were recruited through research registries, past participation in TBI-related studies, and through community organizations serving persons with TBI.

### Ethics approval and consent to participate.

All participants provided informed consent and all procedures were approved by the University of Pittsburgh Institutional Review Board and the University of Texas Southwestern Medical Center Institutional Review Board and performed in accordance with relevant guidelines and regulations.

### Primary Measure: Behavioral Assessment Screening Tool (BAST)

Previous studies report details about the development and psychometric evaluation of the BAST [18–21]. Briefly, the BAST is a multidimensional self-reported neurobehavioral symptom measure assessing frequency of experiencing a symptom or behavior over the past two weeks, ranging from never to very often. The BAST subscales cover five domains: Negative Affect, Fatigue, Executive Dysfunction, Substance Use, and Impulsivity.

### Measures for Convergent, Discriminant, and Known Groups Validity

Participants in the first cohort completed all questionnaires remotely using paper-pencil forms, then returned completed measures via mail in prepaid and addressed envelopes. Participants in the second cohort completed all questionnaires electronically via REDCap^™^. Table 2 summarizes all measures collected for the current analyses, including validated measures of positive and negative affect, depression, anxiety, fatigue, aggression, alcohol misuse, and dysregulated behavior.

To characterize known-groups for depression and anxiety, we classified individuals based on a cut-off score of ≥10 on the Patient Health Questionnaire and on the Generalized Anxiety Disorder-7 to indicate moderate-severe depressive and anxiety symptoms, respectively [22,23]. To characterize known-groups for probable alcohol misuse (only in cohort 2), we classified males with AUDIT scores ≥7 and females with AUDIT scores ≥5 as being indicative of problematic drinking, based on established cut-off scores [24]. To characterize known-groups for fatigue, we classified individuals based on established cut-off scores for PROMIS Fatigue 7-item short form equivalent to a t-score <60 (<23 raw score) vs ≥60 ( ≥23 raw score) in the second cohort [25]. To characterize known-groups for impulsivity and for executive dysfunction, we classified individuals based on established cut-off scores for FrSBe Disinhibition and Executive Function subscales (t-scores <65 vs ≥65) in the first cohort [26].

### Data analysis

We examined descriptive statistics, including frequencies and percentages for demographic characteristics and means and standard deviations for clinical characteristics of the sample. We evaluated convergent and discriminant validity evidence for the BAST subscales using Spearman correlation coefficients examining hypothesized correlations patterns (see Table 1). A pattern of stronger correlations supported between BAST subscales and measures of similar constructs and weaker correlations between BAST subscales and measures of dissimilar constructs would support our hypotheses for convergent and discriminant validity [27].

To examine known-groups validity, we examined differences in BAST subscale scores between those with and without moderate-severe depressive symptoms, moderate-severe anxiety symptoms, clinically significant fatigue, and alcohol abuse using t-tests and Cohen’s d effect sizes. Given the number of analyses, we set a conservative threshold of p<.001 for statistical significance. All statistical analyses were conducted using SPSSv26 for Windows.

## Results

### Participants

Table 3 presents characteristics of the two cohort samples and the number of participants who completed other study measures. Missing measures were either not completed or had missing items preventing valid scoring of that measure. Participants were predominantly White, and a large proportion of participants had a college education in both cohorts. Participants reported experiencing neurobehavioral symptoms, on average, rarely (score of 2) to sometimes (score of 3), except for Substance Use which was reported never to rarely (scores of 1-2). Participants on average also reported clinically significant frontal behaviors (FrSBe t-scores ≥65), fatigue, mild depressive symptoms (PHQ9 scores 5-9), and moderate anxiety (GAD7 scores ≥10).

### Convergent and Discriminant validity

Table 4 presents correlations of the BAST subscales with other validated measures. The BAST subscales generally correlated as hypothesized to support convergent and discriminant validity, with all correlations between BAST subscales and similar measures being stronger than the relationships between BAST subscales and dissimilar measures.

### Known-groups validity

Mean subscales scores between known-groups are presented in Table 5 for each subscale, along with statistical significance of independent t-tests and associated Cohen’s d effect sizes. Negative Affect significantly (p<.001) differentiated those with depression and anxiety, in both cohorts, from those without, with large effect sizes (d=1.4-1.9). Fatigue significantly (p<.001) differentiated those with clinically significant fatigue from those without, with a large effect size (d=−1.8). Substance Use significantly (p<.001) differentiated those with likely alcohol abuse from those without, with a large effect (d=−1.8). Impulsivity significantly (p<.001) differentiated those with Disinhibition from those without, with a large effect (d=1.2). Finally, Executive Dysfunction significantly differentiated those with clinically significant frontal executive disruptions, with a large effect (d=1.2). Notably, the BAST subscales hypothesized to differentiate the known groups had the largest effect sizes (compared to other subscales not hypothesized to differentiate groups), with one exception; for those with frontal executive disruptions as measured by the FrSBe Executive function scale, the BAST Fatigue subscale showed a larger difference (d=1.9) than Executive Dysfunction.

## Discussion

This study further supports the validity of the BAST by demonstrating the construct validity evidence of each of its five subscales, building on the existing evidence of its content validity [18,20], multidimensional factor structure [6], and strong internal consistency reliabilities [19]. The magnitude of the relationships between BAST subscales and measures of similar constructs (including depression, anxiety, positive and negative affect, apathy, fatigue, aggression, disinhibition, executive function, and substance misuse) provided evidence of convergent and discriminant validity as hypothesized.

Including commonly used assessments (PHQ-8, GAD-7, AUDIT, PROMIS, FrsBe) to create known group comparisons, we found that expected subscales of the BAST can differentiate groups with and without a potential clinical condition of interest. Given the hallmark symptoms of depression – poor mood, sleep disruption and fatigue, and changes in thinking and concentration – it makes sense that the Negative Affect, Fatigue, and Executive Dysfunction subscales would differ between those with moderate-severe depressive symptoms from those without. Similarly, the Negative Affect subscale covers the hallmark symptoms of anxiety (worry, agitation), and we found it had the largest effect size across all subscales for differences in those with and without anxiety. Differences in Fatigue, Executive Dysfunction, and Impulsivity were also noted, but with smaller effects. This may be partially due to the high correlations between anxiety and depression after TBI [28]. The Fatigue BAST subscale differentiated the groups with and without fatigue best, but there were also significant differences on Negative Affect, Impulsivity, and Executive Dysfunction, which is not surprising given the hypothesized related constructs. All BAST subscales differed between those scoring low and high on the FrSBe disinhibition scale, with Executive Function and Impulsivity showing the largest effect sizes. While Impulsivity is hypothesized to show this differentiation, the effect of Executive Function may be driven by item overlap measuring empathy and behavioral constraint in social situations. The Substance Use subscale was the only one to differentiate those with and without alcohol abuse as measured by the AUDIT, suggesting it may be an effective short screener for identifying need for further evaluation for substance use disorders. Further work is needed to explore differences in those with clinically significant fatigue, executive dysfunction, and impulsivity, as the measures used to established known groups for these constructs are not well-validated diagnostic screeners like the PHQ9, GAD7, and AUDIT. This may partially explain why the BAST Fatigue scale showed a larger effect for differentiating those with FrSBe-determined executive dysfunction than the BAST Executive Function subscale did. Another example for this finding is that cognitive fatigue can exacerbate the functional consequences of neuropsychological deficits, making fatigue and executive function symptoms difficult to separate [29]. Additionally, further work is required to identify meaningful cut-off scores on the BAST subscales that could inform the need for further clinical evaluation.

### Limitations and Future Directions

Psychometric validation of a tool is an iterative process, and as such, replication and validation studies are necessary as measurement tools develop and are applied to new populations. Future validation studies of the BAST should address some of the present study’s limitations. Most notably, both samples in this study were almost exclusively white and non-Hispanic, with high levels of education, and geographic representation of sample participants was limited. Self-reporting on the BAST may differ based on factors such as race, ethnicity, education, gender, and geographic location [30]. Applying these findings to other English-speaking community-dwelling adults with TBI should be done with careful attention. These data also came from the same studies used to refine the BAST items and factor structure and are therefore prone to the same sampling biases; confirmation of these psychometrics properties in future studies samples is recommended. Refinement to improve the psychometric properties of the BAST is ongoing; however, the BAST continues to demonstrate strong psychometric properties for use as a self-reported neurobehavioral symptom screening measure in chronic TBI.

## Conclusions

The BAST is patient-centered and likely to be well accepted by respondents [18,19]. Its multidimensional nature makes it well suited to assess a broad range of neurobehavioral problems, allowing for more parsimonious measurement over a combination of previously validated measures focused on specific symptoms. Perhaps most importantly, the BAST was specifically developed and tested as a self-reported measure for remote symptom reporting, supporting its incorporation into telehealth and mobile health platforms that can improve chronic symptom monitoring in community-dwelling adults with TBI. With further validation research, behavioral profiles provided by the BAST could be used to predict other rehabilitation outcomes (e.g., community participation, mental health diagnoses, satisfaction with life) and allow for early identification of subgroups of persons with TBI that could benefit from intervention.

## Figures and Tables

**Table 1.** Convergent and Discriminant Validity Measures and Hypothesized Correlations with BAST Subscales

		BAST subscales				
		Hypothesized Correlations				

	Measure	Negative Affect	Substance Use	Executive Dysfunction	Fatigue	Impulsivity
**Both**	**Patient Health Questionnaire 9**	X	O	X	X	O

**Both**	**General Anxiety Disorders 7**	X	O	X	O	X

**Both**	**Positive and Negative Affect Schedule**					
	Positive Affect	X	O	X	X	O
	Negative Affect	X	O	O	X	X

**C1**	**Frontal Systems Behavior Scale**					
	Apathy	X	O	X	X	O
	Disinhibition	X	O	X	O	X
	Executive Dysfunction	X	O	X	X	X

**C1**	**Fatigue Impact Scale**					
	Physical Fatigue	O	O	O	X	O
	Cognitive Fatigue	X	O	X	X	O

**C2**	**PROMIS Fatigue**	O	O	O	X	O

**C1**	**Aggression Questionnaire**					
	Physical Aggression	O	O	O	O	X
	Verbal Aggression	O	O	O	O	X
	Anger Aggression	X	O	X	O	X
	Hostility Aggression	X	O	X	O	X

**C2**	**Alcohol Use Disorders Test**		**Strong**			

X=hypothesized to correlate and demonstrating convergent validity; O=hypothesized to have weak or no correlation demonstrating discriminant validity. C1= Cohort 1 (n=111); C2= Cohort 2 (n= 134). BAST=Behavioral Assessment Screening Tool

**Table 2.** Measures Description

Measure	Description
**Collected in Both Cohorts**	
**Behavioral Assessment Screening Tool**	A 47-item (41 primary, 6 sub-items) self-reported measure to assess neurobehavioral symptoms in chronic brain injury. The BAST has five subscales: Negative Affect, Executive Dysfunction, Fatigue, Impulsivity, & Substance Use. Items are each rated on a 1–5 point ordinal scale indicating frequency of an experience or symptom. Items within each subscale are totaled and divided by the number of items in the subscale to create an average score, indicating never experiencing symptoms (1) to experiencing symptoms always (cohort 1)/very often (cohort 2) (5) in that domain. The BAST demonstrates good content validity and internal consistency reliabilities and a multidimensional factor structure (1–5).
**Positive and Negative Affect Schedule**	A measure of affect that consists of two 10-item subscales: Positive Affect and Negative Affect. Items are rated on a 1-5 scale and summed to yield a total score per subscale (6). Higher scores on the Positive Affect scale indicate high energy, concentration, and pleasurable engagement, whereas low scores indicate sadness and lethargy. Higher scores on the Negative Affect scale indicate high anger, disgust, guilt, fear, or nervousness, whereas low scores indicate calmness and serenity.
**Patient Health Questionnaire 9**	A measure of depressive symptoms specifically validated for use after TBI (7). It includes nine questions rated on a 0–3 point scale. Summed scores indicate depressive symptoms:0-4=None; 5-9=Mild; 10-14=Moderate; 15-19=Moderately Severe; 20-27=Severe (8). For the purposes of assessing known groups validity, we used a cut-off score of ≥10 to indicate at least moderate depressive symptoms.
**General Anxiety Disorder 7**	A measure of general anxiety symptoms (9). It includes seven items rated on a 0-3 point scale, with summed scores indicating severity: 0-4=None; 5-9=Mild; 10-14=Moderate; 15+=Severe (9). For the purposes of assessing known groups validity, we used a cut-off score of ≥10 to indicate at least moderate anxiety symptoms.
**Collected in Cohort 1 only**	
**Fatigue Impact Scale**	A measure of the impact of fatigue on everyday life that has been validated for use after TBI (10,11). We used the Rasch-based Physical (8 items) and Cognitive (5 items) Fatigue scores to describe fatigue in the sample (12).
**Frontal Systems Behavior Scale**	A measure of behavioral changes associated with frontal lobe damage (13–15). The FrSBe has 46 items yielding three subscales of behavioral disruption after TBI: Disinhibition, Apathy, and Executive Function (t-scores). We used the Self-Report form “After Injury” (current) behavior scores. It is a Common Data Element for post-TBI behavior (16).
**Aggression Questionnaire**	A self-reported measure of aggression, including subscales for physical aggression, verbal aggression, anger, and hostility (17,18). Twenty-nine items are rated on a 1-7 point ordinal scale, with higher scores indicating higher levels of aggression in each subscale domain.
**Collected in Cohort 2 only**	
**PROMIS Fatigue**	A 7-item self-reported measure of fatigue over the past week. Each item on this short-form is rated on a 5-level ordinal scale, with higher scores indicating more fatigue (19).
**Alcohol Use Disorders Test**	A 10-item screening tool for alcohol use behaviors that assesses consumption, drinking behaviors, and alcohol-related problems (20).

**Table 3:** Participant Demographic and Clinical Characteristics

	Cohort 1 n	Mean (SD) or n (%)	Cohort 2 n	Mean (SD) or n (%)
Age	110	48.5 (14.5)	134	44.3 (15.7)

Time post-injury (years)	110	8.6 (6.8)	132	7.6 (11.0)

Injury Severity*				
Mild	82	16 (19.5%)		99 (74.5%)
Moderate		11 (13.4%)	133	7 (5.3%)
Severe		55 (67.1%)		27 (20.3%)

Gender				
Women	110	41 (37.3%)	133	78 (58.6%)
Men		69 (62.7%)		54 (40.6%)
Transgender/Other gender		0		1 (0.8%)

Race^≠^				
White	110	102 (92.7%)		114 (85.1%)
Black		7 (6.4%)	134	11 (8.2%)
Other		1 (0.9%)		10 (7.4%)

Education				
High School education	110	59 (53.6%)		45 (33.6%)
Undergraduate degree		39 (35.5%)	134	54 (41.3%)
Graduate degree		12 (10.9%)		36 (26.0%)

BAST: Neurobehavioral Symptoms				
Negative Affect	110	2.7 (.8)	134	3.2 (.8)

Substance Use	110	1.3 (.7)	134	1.3 (.6)

Executive Dysfunction	110	2.2 (.6)	134	2.5 (.7)

Fatigue	110	2.6 (.8)	134	3.6 (.9)

Impulsivity	110	2.1 (.7)	134	2.2 (.7)

Depressive symptoms (PHQ-9/8)	104	7.1 (6.0)	132	9.6 (5.7)

Anxiety (GAD7)	108	12.2 (5.3)	130	13.8 (5.5)

Positive Affect (PANAS)	107	32.3 (8.6)	131	30.0 (9.3)

Negative Affect (PANAS)	107	17.9 (7.4)	132	22.6 (8.7)

Frontal Behaviors [FrSBe (t-scores)]			n/a	n/a
Apathy	102	61.2 (19.7)		
Disinhibition	102	65.2 (18.6)		
Executive Function	102	66.5 (20.3)		

Aggression (AQ)			n/a	n/a
Physical	104	48.4 (9.9)		
Verbal	104	50.7 (10.9)		
Anger	104	49.8 (11.5)		
Hostility	104	52.4 (10.8)		

Fatigue (mFIS)			n/a	n/a
Physical Fatigue	108	21.1 (8.8)		
Cognitive Fatigue	108	12.6 (5.8)		

PROMIS Fatigue	n/a	n/a	130	21.8 (5.8)

Alcohol Abuse (AUDIT)	n/a	n/a	134	2.5 (4.6)

BAST=Behavioral Assessment Screening Tool; FrSBe=Frontal Systems Behavior Scale; PHQ-9=Patient Health Questionnaire-9; GAD-7=Generalized Anxiety Disorder-7; PANAS=Positive and Negative Affect Schedule; mFIS=modified Fatigue Impact Scale; AQ=Aggression Questionnaire; AUDIT=Alcohol Use Disorders Test

* Injury severity based on OSU-TBI worst injury score. Though TBI was confirmed through either OSU-TBI or inclusion in a previous study requiring medical documentation of TBI, the OSU-TBI was not collected for all participants.

≠ Multiple races could be selected; numbers indicate how many participants selected that race, regardless of other races they selected.

**Table 4:** Convergent and Discriminant Correlations with BAST Subscales

	Negative Affect	Substance Use	Executive Dysfunction	Fatigue	Impulsivity
**Cohort 1 and 2** (Cohort 1 correlation coefficients on top; Cohort 2 correlation coefficients on bottom)					
**Depression (PHQ8)**	**.684** * **.757** *	.137 .116	.617* .547*	**.674** * **.646** *	.399* .373*
**Anxiety (GAD7)**	**.813** * **.770** *	.271* .183	.475* .366*	.478* .430*	.435* .381*
**Positive Affect (PANAS)**	−.440* −.441*	.008 .098	−.587* −.603*	−.527* −.601*	−.144 −.236
**Negative Affect (PANAS)**	**.782** * **.767** *	.353* .006	.377* .364*	.470* .364*	.460* .388*
**Cohort 1 Only**					
**Apathy (FrSBe)**	.587*	0.152	**.622** *	**.605** *	.271*
**Disinhibition (FrSBe)**	.589*	.375*	.489*	.430*	.577*
**Executive Function (FrSBe)**	**.601** *	.211	**.683** *	.508*	.533*
**Physical Aggression (AQ)**	.287*	.374*	.251	.175	.305*
**Verbal Aggression (AQ)**	.261*	.248	.256*	.103	.278*
**Anger Aggression (AQ)**	**.602** *	.319*	.468*	.264*	.458*
**Hostility Aggression (AQ)**	.510*	.289*	.351*	.364*	.398*
**Physical Fatigue (MFIS)**	.485*	0.136	.505*	**.828** *	.291*
**Cognitive Fatigue (MFIS)**	**.688** *	.212	.543*	**.726** *	.482*
**Cohort 2 Only**					
**Alcohol Abuse (AUDIT)**	.123	**.721** *	−.053	−.102	.151
**Fatigue (PROMIS)**	.584*	−.038	.539*	**.815** *	.294*

Lighter shading: hypothesized medium correlations; Darker shading: hypothesized strong correlations

Medium r >.30 to .60; **Strong r >.60**

* p<.001 (2-tailed)

**Table 5:** BAST subscale differences by likely depression and anxiety

	Depression	No Depression		

BAST Subscale	Mean (SD)	Mean (SD)	*p*	*d*
**Cohort 1**	**n=30**	**n=74**		
Negative Affect	3.36 (.59)	2.39 (.64)	<.001	1.5

Substance Use	1.41 (.73)	1.27 (.59)	.307	0.2

Executive Dysfunction	2.72 (.54)	1.96 (.45)	<.001	1.6

Fatigue	3.43 (.56)	2.33 (.72)	<.001	1.6

Impulsivity	2.54 (.75)	1.90 (.65)	<.001	0.9

**Cohort 2**	**n=61**	**n=71**		
Negative Affect	3.72 (.66)	2.72 (.60)	<.001	1.6

Substance Use	1.36 (.68)	1.26 (.46)	.307	0.2

Executive Dysfunction	2.80 (.57)	2.19 (.68)	<.001	1.0

Fatigue	4.06 (.65)	3.15 (.91)	<.001	1.1

Impulsivity	2.46 (.79)	2.08 (.65)	<.001	0.5
	Anxiety	No Anxiety		

BAST Subscale	Mean (SD)	Mean (SD)		

**Cohort 1**	**n=62**	**n=46**		
Negative Affect	3.15 (.61)	2.07 (.48)	<.001	1.9

Substance Use	1.49 (.83)	1.13 (.34)	.003	0.5

Executive Dysfunction	2.37 (.55)	1.91 (.54)	<.001	0.9

Fatigue	2.91 (.78)	2.28 (.76)	<.001	0.8

Impulsivity	2.30 (.66)	1.81 (.74)	<.001	0.7

**Cohort 2**	**n=98**	**n=32**		
Negative Affect	3.42 (.72)	2.43 (.55)	<.001	1.4

Substance Use	1.32 (.60)	1.22 (.44)	.365	0.2

Executive Dysfunction	2.62 (.66)	1.98 (.59)	<.001	1.0

Fatigue	3.75 (.85)	2.99 (.91)	<.001	0.9

Impulsivity	2.36 (.73)	1.92 (.69)	.003	0.6
	Fatigue	No Fatigue		

BAST Subscale	Mean (SD)	Mean (SD)		

**Cohort 2**	**n=65**	**n=65**		
Negative Affect	3.62 (.71)	2.77 (.66)	<.001	1.2

Substance Use	1.33 (.63)	1.28 (.52)	.614	0.1

Executive Dysfunction	2.77 (.65)	2.17 (.63)	<.001	0.9

Fatigue	4.19 (.66)	2.97 (.72)	<.001	1.8

Impulsivity	2.44 (.77)	2.07 (.67)	.004	0.5
	Alcohol Abuse	No Abuse		

BAST Subscale	Mean (SD)	Mean (SD)		

**Cohort 2**	**n=21**	**n=113**		
Negative Affect	3.28 (.83)	3.17 (.80)	.565	0.1

Substance Use	2.02 (.95)	1.17 (.33)	<.001	1.8

Executive Dysfunction	2.28 (.69)	2.50 (.69)	.185	0.3

Fatigue	3.19 (1.0)	3.66 (.88)	.033	0.5

Impulsivity	2.38 (.79)	2.22 (.73)	.365	0.2
	Disinhibition	No Disinhibition		

BAST Subscale	Mean (SD)	Mean (SD)		

**Cohort 1**	**n=38**	**n=64**		
Negative Affect	3.14 (.73)	2.43 (.68)	<.001	1.0
Substance Use	1.64 (.98)	1.18 (.39)	.008	0.7
Executive Dysfunction	2.53 (.62)	1.95 (.45)	<.001	1.1
Fatigue	3.02 (.71)	2.42 (.81)	<.001	0.8
Impulsivity	3.02 (.71)	1.83 (.67)	<.001	1.2
	Executive Dysfunction	No Executive Dysfunction		

BAST Subscale	Mean (SD)	Mean (SD)		

**Cohort 1**	**n=48**	**n=54**		
Negative Affect	3.08 (.67)	2.35 (.71)	<.001	1.1
Substance Use	1.42 (.77)	1.29 (.64)	.369	0.2
Executive Dysfunction	2.48 (.59)	1.89 (.42)	<.001	1.2
Fatigue	3.02 (.71)	2.31 (.77)	<.001	1.9
Impulsivity	2.47 (.69)	1.80 (.65)	<.001	1.0
